# Standardized Assessment of Resistance Training-Induced Subjective Symptoms and Objective Signs of Immunological Stress Responses in Young Athletes

**DOI:** 10.3389/fphys.2018.00698

**Published:** 2018-06-05

**Authors:** Christian Puta, Thomas Steidten, Philipp Baumbach, Toni Wöhrl, Rico May, Michael Kellmann, Marco Herbsleb, Brunhild Gabriel, Stephanie Weber, Urs Granacher, Holger H. W. Gabriel

**Affiliations:** ^1^Department of Sports Medicine and Health Promotion, Friedrich-Schiller-University Jena, Jena, Germany; ^2^Department of Anesthesiology and Intensive Care Medicine, University Hospital Jena, Jena, Germany; ^3^Sportgymnasium Jena “Johann Chr. Fr. GutsMuths”, Jena, Germany; ^4^Faculty of Sport Science, Ruhr University, Bochum, Germany; ^5^School of Human Movement and Nutrition Sciences, The University of Queensland, St Lucia, QLD, Australia; ^6^Division of Training and Movement Sciences, Research Focus Cognition Sciences, University of Potsdam, Potsdam, Germany

**Keywords:** immune system, strength training, track and field, youth, Acute Recovery and Stress Scale (ARSS)

## Abstract

From a health and performance-related perspective, it is crucial to evaluate subjective symptoms and objective signs of acute training-induced immunological responses in young athletes. The limited number of available studies focused on immunological adaptations following aerobic training. Hardly any studies have been conducted on resistance-training induced stress responses. Therefore, the aim of this observational study was to investigate subjective symptoms and objective signs of immunological stress responses following resistance training in young athletes. Fourteen (7 females and 7 males) track and field athletes with a mean age of 16.4 years and without any symptoms of upper or lower respiratory tract infections participated in this study. Over a period of 7 days, subjective symptoms using the Acute Recovery and Stress Scale (ARSS) and objective signs of immunological responses using capillary blood markers were taken each morning and after the last training session. Differences between morning and evening sessions and associations between subjective and objective parameters were analyzed using generalized estimating equations (GEE). In *post hoc* analyses, daily change-scores of the ARSS dimensions were compared between participants and revealed specific changes in objective capillary blood samples. In the GEE models, recovery (ARSS) was characterized by a significant decrease while stress (ARSS) showed a significant increase between morning and evening-training sessions. A concomitant increase in white blood cell count (WBC), granulocytes (GRAN) and percentage shares of granulocytes (GRAN_%_) was found between morning and evening sessions. Of note, percentage shares of lymphocytes (LYM_%_) showed a significant decrease. Furthermore, using multivariate regression analyses, we identified that recovery was significantly associated with LYM_%_, while stress was significantly associated with WBC and GRAN_%_. *Post hoc* analyses revealed significantly larger increases in participants’ stress dimensions who showed increases in GRAN_%_. For recovery, significantly larger decreases were found in participants with decreases in LYM_%_ during recovery. More specifically, daily change-scores of the recovery and stress dimensions of the ARSS were associated with specific changes in objective immunological markers (GRAN_%_, LYM_%_) between morning and evening-training sessions. Our results indicate that changes of subjective symptoms of recovery and stress dimensions using the ARSS were associated with specific changes in objectively measured immunological markers.

## Introduction

According to Nieman (1994), high training volumes and intensities are associated with a higher risk of sustaining respiratory tract infections. This could be due to acute immunological responses which are often observed after intense training sessions (Northoff et al., 1998). From a health and performance-related perspective, it is important to detect and evaluate training-induced symptoms and signs of immunological responses in young athletes. Previous studies examined training-induced immunological responses in child and adolescent athletes (see **Table 1**). For instance, Freitas et al. (2016) monitored 26 young male soccer players (15.6 ± 1.1 years) and observed differences in immunological markers between official and simulated soccer matches. The evaluation of 15 mL salvia samples revealed a significant saliva immunoglobulin A (s-IgA) reduction after official matches. There is evidence that different s-IgA kinetics are associated with higher ratings of perceived exertion (RPE) in official matches which is indicative of a relation between intensity and immune response (Freitas et al., 2016; Owen et al., 2016). Interestingly, Moraes et al. (2017) demonstrated that an intensified training load followed by a tapering period negatively affected the mucosal immune function in young basketball players without causing significant changes in severity of upper respiratory tract infections. Of note, findings from Moraes et al. (2017) are in line with data from Gleeson et al. (2000) who evaluated markers of immune function over a 12-week training period in 22 elite swimmers aged 16–22 years. These authors did not detect significant associations between serum or salivary immunoglobulin levels, NK-cell numbers and symptoms of upper respiratory tract illness over the course of the 12-week training program (Gleeson et al., 2000). In addition, Moreira et al. (2013) did not find significant changes in objectively measured immunological parameters (s-IgA) after a 4-week training program in 12 male elite futsal players. In contrast, results from Mortatti et al. (2012) indicate that decrements in mucosal immunity (s-IgA) lead to a greater incidence of upper respiratory tract infections in elite young soccer players. Furthermore, these authors were able to establish a link between upper respiratory infections (URTI) and immunological responses (Mortatti et al., 2012) and between RPE and URTI (Moreira et al., 2013).

**Table 1:** Mini-Review of immunological responses in young athletes.

Publication	Subjects	Method	Main measures	Period	ISR?	Acute or chronic?
Freitas et al., 2016	*N* = 26 male youth soccer players (15.6 ± 1.1 years)	15 ml salvia samples	s-IgA	1 week (2 official and 2 simulated matches)	yes	acute
Moraes et al., 2017	*N* = 23 male youth basketball players (15.8 ± 0.8 years)	15 ml salvia samples, WURSS-21, Borg-Scale	s-IgA, URTI, RPE	8 weeks training (1 familiarization, 4 intensive training, 3 taper)	yes	chronic
Mortatti et al., 2012	*N* = 14 elite male youth soccer players (18.5 ± 0.4 years)	15 ml salvia samples, Borg-Scale	s-IgA, Cortisol, RPE	20 days (7 matches)	yes	chronic
Gleeson et al., 2000	*N* = 22 elite swimmers (16–22 years, 12 m/10f)	1 mL salvia, 2 mL blood samples	s-IgA, s-IgM, s-IgG, NK-Cells, Albumin	12 weeks training	yes	chronic
Moreira et al., 2013	*N* = 12 male elite futsal players (19.0 ± 1 years)	15 mL saliva samples, WURSS-21, Borg	s-IgA, URTI, RPE	4 weeks training	no	chronic

*WURSS-21, Wisconsin Upper Respiratory Symptom Survey-21; s-IgA, secretory immunoglobuline A; s-IgM, secretory immunoglobuline M; s-IgG, secretory immunoglobuline G; NK-Cells, natural killer cells; URTI, upper respiratory infections; RPE, rating of perceived exertion; ISR, training-induced immunological responses*.

Training responses can also be monitored using self-reported questionnaires. Thereby, athletes’ perceived internal load, mood and recovery-stress states can be examined. Previously, it has been shown that mood disturbances are associated with performance declines and biological changes such as immunosuppression (Fry et al., 1994).

Spence et al. (2007) reported that elite athletes suffer more often from URTI symptoms than recreational athletes. Notably, URTI symptoms caused by infections were registered in 30% of all examined athlete. No causes of URTI symptoms were identified in the remaining 70% of athletes. Interestingly, both infected and unidentified athletes showed similar symptoms during the first 2 days (Spence et al., 2007). The results from Spence et al. (2007) imply the need to evaluate objective and subjective measures to distinguish between infect-based and exercise-induced immunological responses.

Walsh et al. (2011) in their position statement recommended that “we need to focus on the nature of exercise.” Most studies in the field of “exercise and immune response” are related to cardiorespiratory exercises (e.g., endurance, aerobic training). In contrast, the effects of resistance training on pro- and anti-inflammatory processes remains poorly understood (Walsh et al., 2011) which is why this is an area for future investigations.

Therefore, the aim of this observational study was to examine resistance-training induced changes in subjective [i.e., Acute Recovery and Stress Scale (ARSS)] and objective measures (i.e., capillary blood) of immunological stress responses over a period of 7 days with daily morning and evening tests in young athletes. In addition, potential associations between subjective and objective markers of immunological stress responses were computed. Recently, Kellmann et al. (2016) developed ARSS to asses and monitor multidimensional recovery and stress states. ARSS represents a promising assessment tool that considers multidimensionality and sport specificity. Most importantly, the ARSS questionnaire is economically valid (Kellmann et al., 2016).

With reference to the relevant literature on ARSS (Kölling et al., 2015; Kellmann et al., 2016; Hitzschke et al., 2017), we expected that stress dimensions increase and recovery dimensions decrease from morning to evening training sessions. In accordance with findings from Ihalainen et al. (2014), we hypothesized that granulocytes would increase from morning to evening training sessions because young athletes are subject to endocrine, immunological, and metabolic stress due to school, training, and social demands. Given that subjective and objective measures are sensitive to strains of everyday life and training, we hypothesized that changes in subjective symptoms using ARSS between morning and evening training sessions are associated with changes in objective signs following resistance training-induced immunological responses in young track and field athletes.

## Materials and Methods

### Participants

Our study sample was recruited with reference to the recently introduced conceptual model on how to implement resistance training during the stages of long-term athlete development (Granacher et al., 2016). According to this model, young athletes aged between 12 and 18 years (Granacher et al., 2016) who span the stages “Late Childhood” (pre-pubertal, Tanner Stage I-II) and “Adolescents” (pubertal, Tanner Stage III-IV) would participate in regular resistance training programs. Thus, 14 (7 females and 7 males) young track and field athletes with a mean age of 16.4 years and an age range of 15–18 years participated in this 7-day observational study. To be eligible for inclusion in this study, athletes had to be free from any signs and symptoms of upper or lower respiratory tract infections during the last 2 weeks prior to the start of this study. If any sign or symptoms (>48 h) occurred during the 7-day observational study, participants were excluded from our final statistical analyses (Spence et al. (2007). In addition, participating athletes had to be experienced with resistance training (minimum 2 years of experience) and conduct resistance training over the 7-day study period. Track and field athletes were recruited because the multidimensional demands of this sport afford resistance training for performance development and injury prevention. Our study sample was experienced with free weight training, core strength training, and plyometric training and performed these types of resistance training during their daily training routines. Prior to the start of the study, experimental procedures, risks and benefits of the study were explained to all participating athletes and their legal representatives and written informed consent was obtained from all involved parties. This study was carried out in accordance with the recommendations of the University Research Ethics Committee of the Friedrich-Schiller-University Jena, Germany and the latest version of the Declaration of Helsinki. The protocol was approved by the University Research Ethics Committee of the Friedrich-Schiller-University Jena (458510/15).

### Contents of Training

Training was performed in small athletic groups (2–3) or even individually according to age of participants and sport discipline. Due to the multidimensional demands of track and field disciplines, resistance training sessions addressed neuromuscular adaptations using core strength training, plyometric training, and free weight training. In addition, technical aspects of resistance training to achieve sufficient movement quality was part of the program as well (see Supplementary Material for detailed information).

### Procedures

Subjective and objective measures were taken each morning and after the last training session of the day. Subjective symptoms of immunological stress responses were assessed using ARSS. Objective markers were taken from capillary blood samples to evaluate immune cell distribution. In order to exclude *ad hoc* infectious cases from our analyses, a questionnaire was used to identify signs of upper and lower respiratory infections (ULI).

#### Subjective Measures

The German version of the ARSS (Kölling et al., 2015; Hitzschke et al., 2016; Kellmann et al., 2016) was used as an assessment tool to record recovery and stress states in our study sample. The English version of the ARSS was recently described and published elsewhere (Nässi et al., 2017). ARSS consists of 32 items and it assesses aspects of emotional, physiological, mental, and overall stress and recovery. Those items are equally arranged and distributed in four stress-related (i.e., muscular stress, lack of activation, negative emotional state, and overall stress) and four recovery-related (physical performance capability, mental performance capability, emotional balance, and overall recovery) scales (Kellmann et al., 2016). Participants were asked to rate each item on a seven-point Likert-type scale from 0 (does not apply at all) to 6 (fully applies). Several recently published studies (Kölling et al., 2015; Kellmann et al., 2016; Hitzschke et al., 2017) indicated that the ARSS is a sensitive measure for the evaluation of recovery and stress states in athletes. All scales of the ARSS showed satisfactory internal consistency (range between α = 0.84 and α = 0.96) and a good model fit for both the recovery (RMSEA = 0.07, CFI = 0.97, SRMR = 0.04) and stress (RMSEA = 0.09, CFI = 0.94, SRMR = 0.05) items (Kellmann et al., 2016). These dimensions were used for further analysis.

#### Objective Measures

The capillary blood markers white blood cells (WBC), Lymphocytes (LYM), Lymphocytes % (LYM_%_), Monocytes (MID) Monocytes % (MID_%_), Granulocytes (GRAN), Granulocytes % (GRAN_%_), red blood cells (RBC), Hemoglobin (HGB), Hematocrit (HCT), mean corpuscular volume (MCV), mean corpuscular concentration (MCVC), red blood distribution width (RDW_%_), red blood cell distribution width absolute (RDWa), Platelets (PLT), platelet distribution width (PDW, fl), Large platelet (LPCR, %) were measured using a 20 μl capillary blood sample taken from the earlobe between 30 and 45 min after morning and evening training sessions. Analysis was performed using a medonic hematology system (Medonic M16M, Boule Medical AB, Spånga, Sweden). Intra-Assay Coefficients of Variability for Micro Pipette Adapters were recently provided by the manufacturer (WBC ≤ 2,5%, RBC ≤ 1,5%, MCV ≤ 0,5%, PLT ≤ 3,0%, HGB ≤ 1,3%; Medical AB, Spånga, Sweden). Our own measurements revealed acceptable Intra-Assay Coefficients of Variability for WBC ≤ 2,22%, GRAN ≤ 2,14%, GRAN_%_ ≤ 1,42%, LYM ≤ 2,95%, LYM_%_ ≤ 1,20%. Inter-Assay Coefficients of Variability were also acceptable with WBC ≤ 1,06%, GRAN ≤ 1,40%, GRAN_%_ ≤ 1,39%, LYM ≤ 2,90%, LYM_%_ ≤ 2,11%.

### Statistical Analyses

#### Resistance Training and Immunological Response

Differences between time of day (morning vs. evening training session) in the ARSS dimensions and the capillary blood markers were tested using generalized estimating equations (GEE). This technique is appropriate for repeated measurements (i.e., 7 training days with two tests per day). It allows imputation of randomly missing data for different time points, and it produces robust parameter estimates and standard errors (Zeger and Liang, 1986; Burton et al., 1998). The recorded ARSS dimensions and capillary blood markers served as dependent variables. The factor *time of day* (dichotomous: morning vs. evening training session) was entered as independent variable in our statistical model. We used a Gaussian link function and within-subject dependencies were modeled as first-order autoregressive. Finally, to examine significant differences between *time of day*, we estimated pairwise contrasts of the marginal means. The reported marginal means and corresponding confidence intervals were collected for all training days.

#### Relation of the Acute Recovery and Stress Scale With Capillary Blood Markers

To assess associations between objectively tested capillary blood markers and subjectively recorded recovery and stress ratings, GEE was used as well. All variables were z-transformed before a regression analysis was computed. Here, ARSS dimensions served as dependent variables. In univariate analysis, the capillary blood markers (e.g., percent of lymphocytes) were entered separately as independent variables. In accordance with recommendations for logistic regression analyses (Bursac et al., 2008), all capillary blood markers with a *p*-value ≤ 0.2 in univariate analyses were entered in a multivariate model. In case of highly correlated blood markers (e.g., absolute values and percent of lymphocytes), the parameter with the better univariate model fit (Quasi Likelihood under Independence Model Criterion, QIC) was entered in the multivariate model. Finally linear stepwise regression analyses was used to remove predictors with *p*-values > 0.1 (starting with the highest *p*-value) from the model (Bursac et al., 2008). During each step, the model fit was evaluated by means of the QIC. In case of a worsening of the model-fit, the respective parameter was kept in the final model.

#### Daily Change-Scores of Acute Recovery and Stress Scale and Capillary Blood Samples

Using *post hoc* analyses, we aimed at comparing ARSS recovery and stress dimensions between subjects. For this purpose, participants were selected who showed an increase in GRAN_%_ (vs. none) or a decrease in LYM_%_ (vs. none). In a first step, change-scores were calculated from morning to evening training sessions for all ARSS dimensions and the blood markers. Next, participants were classified in groups who showed an increase in GRAN_%_ or a decrease in LYM_%_. Change-scores of ARSS dimensions were compared between the respective groups using Mann-Whitney-*U*-Tests. In addition, effect sizes were reported as Pearson correlation coefficients (r = 0.5; 0.3; 0.1 corresponding to large, medium, small effects; Fritz et al., 2012).

The significance level was set at α = 5% and we reported two-sided *p*-values. In case of multiple comparisons, Bonferroni-Holm corrected *p*-values were used. Data analyses was computed using SPSS (Version 22, IBM, United States).

## Results

Our final analysis included 56 datasets with complete information for the morning and evening training sessions (112 observations from 13 athletes). In accordance with Spence et al. (2007), one participant (KS008) was excluded from our final data analysis because of showing ULI signs and symptoms for longer than 48 h.

### Resistance Training and Subjective Symptoms

**Table 2** contains marginal means and 95% confidence intervals for ARSS from GEE models. Pairwise comparisons revealed a highly significant decrease (*p* < 0.001) in the ARSS recovery dimension between morning and evening training sessions. In addition, a highly significant increase (*p* < 0.001) in the ARSS stress dimension was noted between morning and evening training sessions.

**Table 2:** Marginal means and confidence intervals (95%CI) resulting from the Generalized Estimating Equation models for the dimensions of the Acute Recovery and Stress Scale (ARSS) and capillary blood markers.

		Morning			Evening			p_raw_	p_adj_
		Mean	95% CI		Mean	95% CI			
ARSS: recovery	[0–5]	4.46	4.10	4.82	4.01	3.67	4.36	**<0.001**	**<0.001**
ARSS: stress	[0–5]	0.91	0.63	1.20	1.51	1.13	1.89	**<0.001**	**<0.001**
									
White blood cells	[10^9^/l]	7.41	6.88	7.95	9.26	8.16	10.36	**<0.001**	**<0.001**
Lymphocytes	[10^9^/l]	2.98	2.64	3.33	3.14	2.79	3.48	0.363	0.999
Lymphocytes	[%]	40.55	37.33	43.76	35.10	32.50	37.71	**<0.001**	**<0.001**
Monocytes	[10^9^/l]	0.63	0.58	0.67	0.75	0.65	0.85	**0.006**	0.063
Monocytes	[%]	7.74	7.39	8.08	7.53	7.20	7.86	0.165	0.999
Granulocytes	[10^9^/l]	3.79	3.43	4.15	5.36	4.59	6.14	**<0.001**	**<0.001**
Granulocytes	[%]	51.69	48.40	54.98	57.34	54.58	60.10	**<0.001**	**<0.001**
Red blood cells	[10^12^/l]	4.66	4.45	4.87	4.52	4.34	4.69	**0.005**	0.062
Hemoglobin	[mmol/l]	8.70	8.34	9.05	8.46	8.15	8.76	**0.004**	0.052
Hematocrit	[%]	38.80	37.33	40.28	37.68	36.42	38.95	**0.003**	**0.049**
Mean corpuscular volume	[fl]	83.22	82.21	84.22	83.33	82.43	84.24	0.450	0.999
MCH	[fmol]	1.87	1.84	1.90	1.87	1.85	1.90	0.382	0.999
MCHC	[mmol/l]	22.47	22.32	22.63	22.49	22.31	22.66	0.661	0.999
Blood cell distribution width	[fl]	52.26	51.29	53.24	52.53	51.58	53.47	0.194	0.999
Red blood cell distribution width	[%]	13.94	13.74	14.14	13.99	13.77	14.21	0.083	0.834
Platelets	[10^9^/l]	136.49	119.34	153.63	137.36	121.61	153.11	0.870	0.999
Platelet distribution width	[fl]	12.72	12.13	13.31	12.91	12.25	13.56	0.205	0.999
Large platelet	[%]	22.05	19.98	24.11	22.60	20.18	25.01	0.250	0.999

*Significant differences between time of day (morning vs. evening training sessions) are marked with bold *p*-values. Raw (p_*raw*_) and Bonferroni-Holm corrected *p*-values are reported (p_*adj*_)*.

### Resistance Training and Objective Signs

Results for capillary blood markers from GEE models are displayed in **Table 2** as well. Pairwise comparisons of capillary blood parameters revealed a highly significant increase (*p* < 0.001) in WBC, GRAN and GRAN_%_ between morning and evening training sessions. In addition, a significant decrease was found for LYM_%_ (*p* < 0.001) and HCT (*p* = 0.049) from morning to evening training sessions. Statistical trends in the form of an increase were found for MID (*p* = 0.063) and RBC (*p* = 0.062) and a decrease for HGB (*p* = 0.052). Of note, changes in WBC, GRAN, GRAN_%_, and LYM_%_ exceeded Intra-Assay and Inter-Assay Coefficients of Variability.

### Associations of the Acute Recovery and Stress Scale With Capillary Blood Markers

Results of the univariate regression analyses for the ARSS dimensions are displayed in **Table 3**. In summary, ARSS recovery ratings were positively associated with LYM (standardized regression coefficient βz = 0.09, 95% CI: -0.03 to 0.22, *p* = 0.127), LYM_%_ (βz = 0.38, 95% CI: 0.18 to 0.59, *p* < 0.001), MID_%_ (βz = 0.17, 95% CI: -0.01 to 0.35, *p* = 0.057), RBC (βz = 0.15, 95% CI: -0.01 to 0.32, *p* = 0.072), HGB (βz = 0.13, 95% CI: -0.04 to 0.30, *p* = 0.124), HCT (βz = 0.12, 95% CI: -0.03 to 0.28, *p* = 0.117) and negatively associated with WBC (βz = -0.21, 95% CI: -0.39 to -0.03, *p* < 0.05), GRAN (βz = -0.29, 95% CI: -0.49 to -0.08, *p* = 0.006), GRAN_%_ (βz = -0.38, 95% CI: -0.58 to -0.18, *p* < 0.001) and RDWa (βz = -0.19, 95% CI: -0.41 to 0.04, *p* = 0.106). Stress ratings were positively associated with WBC (βz = 0.34, 95% CI: -0.14 to 0.55, *p* = 0.001), MID (βz = 0.20, 95% CI: -0.01 to 0.41, *p* = 0.064), GRAN (βz = 0.40, 95% CI: 0.19 to 0.62, *p* < 0.001), GRAN_%_ (βz = 0.40, 95% CI: 0.19 to 0.62, *p* < 0.001) and negatively associated with LYM_%_ (βz = -0.41, 95% CI: -0.62 to -0.19, *p* < 0.001) and MID_%_ (βz = -0.15, 95% CI: -0.34 to 0.04, *p* = 0.123).

**Table 3:** Results of the univariate regression analysis using generalized estimating equations for the dimensions of the Acute Recovery and Stress Scale (ARSS).

		ARSS: stress				ARSS: recovery			
		β	95%CI		*p*	β	95%CI		*p*
White blood cells	*z*-score	0.34	0.14	0.55	**0.001**	-0.21	-0.39	-0.03	**0.020**
Lymphocytes	*z*-score	0.00	-0.11	0.12	0.985	0.09	-0.03	0.22	**0.127^a^**
Lymphocytes: percent	*z*-score	-0.41	-0.62	-0.19	**<0.001**	0.38	0.18	0.59	**0.000**
Monocytes	*z*-score	0.20	-0.01	0.41	**0.064^a^**	-0.09	-0.27	0.08	0.294
Monocytes: percent	*z*-score	-0.15	-0.34	0.04	**0.123**	0.17	-0.01	0.35	**0.057**
Granulocytes	*z*-score	0.40	0.19	0.62	**<0.001^a^**	-0.29	-0.49	-0.08	**0.006^a^**
Granulocytes: percent	*z*-score	0.40	0.19	0.62	**<0.001**	-0.38	-0.58	-0.18	**<0.001**
Red blood cells	*z*-score	-0.08	-0.30	0.15	0.493	0.15	-0.01	0.32	**0.072**
Hemoglobin	*z*-score	-0.08	-0.29	0.12	0.424	0.13	-0.04	0.30	**0.124**
Hematocrit	*z*-score	-0.07	-0.27	0.12	0.468	0.12	-0.03	0.28	**0.117**
Mean corpuscular volume	*z*-score	0.03	-0.25	0.31	0.840	-0.19	-0.50	0.12	0.230
MCH	*z*-score	0.02	-0.30	0.34	0.894	-0.17	-0.45	0.11	0.238
MCHC	*z*-score	-0.02	-0.18	0.15	0.829	0.01	-0.16	0.19	0.868
Blood cell distribution width: absolute	*z*-score	0.10	-0.07	0.27	0.260	-0.19	-0.41	0.04	**0.106**
Red blood cell distribution width: percent	*z*-score	0.10	-0.23	0.43	0.551	-0.09	-0.38	0.20	0.532
Platelets	*z*-score	0.08	-0.24	0.40	0.633	-0.19	-0.48	0.10	0.204
Platelet distribution width	*z*-score	0.00	-0.24	0.24	0.992	-0.08	-0.29	0.14	0.479
Large platelet: percent	*z*-score	-0.02	-0.27	0.23	0.885	-0.05	-0.26	0.17	0.651

^a^*Because of high multicollinearity between absolute and relative values and a better model fit only percent values were entered in the multivariate models. Standardized regression weights* (β) *and corresponding confidence intervals (95%CI) and *p*-values are presented. Bold p-values indicate variables used for multivariate modeling*.

Findings from multivariate regression analyses revealed that recovery ratings were positively associated with LYM_%_ (βz = 0.38, 95% CI: 0.18 to 0.59, *p* < 0.001) and negatively associated with GRAN_%_ (βz = -0.39, 95% CI: -0.59 to -0.19, *p* < 0.001) and RDWa (βz = -0.21, 95% CI: -0.43 to 0.02, *p* = 0.073). Stress ratings were positively associated with WBC (βz = 0.22, 95% CI: 0.06 to 0.38, *p* = 0.006) and GRAN_%_ (βz = 0.31, 95% CI: 0.12 to 0.50, *p* = 0.001) and negatively associated with LYM_%_ (βz = -0.21, 95% CI: -0.51 to -0.11, *p* = 0.002). Due to high multicollinearity, it was not possible to include LYM_%_ and GRAN_%_ in a joint model (see **Table 4**).

**Table 4:** Results of the multivariate regression analysis using Generalized Estimating Equations for the dimensions of the Acute Recovery and Stress Scale (ARSS).

	Unit	Model I				Model II			
		β	95%CI		*p*	β	95%CI		*p*
**ARSS: recovery**									
*Intercept*		*0.00*	*-0.36*	*0.37*	*0.998*	*0.00*	*-0.36*	*0.37*	*0.991*
Lymphocytes: percent	*z*-score	0.38	0.18	0.59	<0.001	was not entered because of high multicollinearity			
Granulocytes: percent	*z*-score	was not entered because of high multicollinearity				-0.39	-0.59	-0.19	<0.001
Blood cell distribution width: absolute	*z*-score	-0.20	-0.43	0.04	0.098	-0.21	-0.43	0.02	0.073
**ARSS: stress**									
*Intercept*		-0.07	-0.47	0.33	0.738	-0.07	-0.47	0.33	0.729
White blood cells	*z*-score	0.22	0.05	0.38	0.010	0.22	0.06	0.38	0.006
Lymphocytes: percent	*z*-score	-0.31	-0.51	-0.11	0.002	was not entered because of high multicollinearity			
Granulocytes: percent	*z*-score	was not entered because of high multicollinearity				0.31	0.12	0.50	0.001

*Standardized regression weights* (β) *and corresponding confidence intervals (95%CI) and *p*-values are presented*.

**FIGURE 1:**
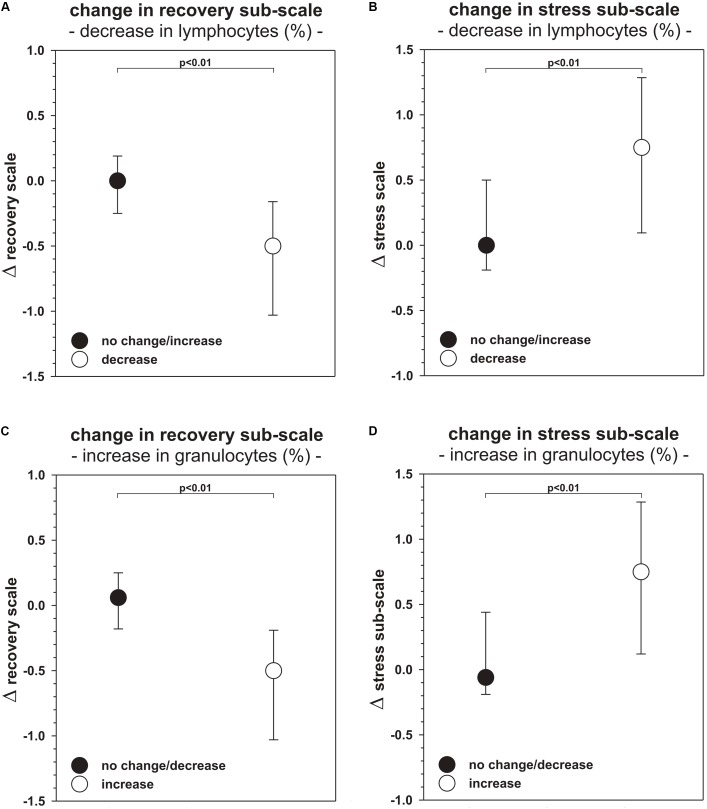
Median and first/third quartile (Q1/3) of the change-scores in the Acute Recovery and Stress Scale stratified by changes in percentage of lymphocytes **(A,B)** and granulocytes **(C,D)**. Bonferroni-Holm corrected *p*-values (p) of the Mann-Whitney-*U*-Tests are presented.

*Post hoc* analyses (see **Figure 1**) revealed that participants who showed a decrease in LYM_%_ (*n* = 41/56 datasets, vs. no change or increase) between morning and evening training sessions reported a significantly higher decrease (*p* < 0.01) in ARSS recovery dimensions (*r* = 0.40). In line with this finding, the aforementioned athletes also showed a significantly higher increase (*p* < 0.01) in the ARSS stress scale (*r* = 0.36). In addition, participants with an increase in GRAN_%_ (*n* = 41/56 datasets vs. no change or decrease) showed a significantly higher decrease (*p* < 0.01) in ARSS recovery dimensions (*r* = 0.47) and a significantly higher increase (*p* < 0.01) in ARSS stress dimensions (*r* = 0.41).

## Discussion

The main goal of this observational study was to examine resistance-training induced changes in subjective (i.e., ARSS) and objective measures (i.e., capillary blood) of immunological stress responses over a period of 7 days with daily morning and evening tests in young track and field athletes. Research on this topic is scarce because most studies examined immunological responses following endurance/aerobic training (Walsh et al., 2011). Our data demonstrated for the first time that resistance training evokes exercise-induced immunological responses in young track and field athletes. From a practitioners point of view, an important finding of this study is that training-induced changes in ARSS stress and recovery dimensions were associated with capillary blood markers. Thus, ARSS can be used as an easy-to-administer tool to detect signs of immunological stress responses.

### Resistance Training and Subjective Symptoms

It has previously been reported that ARSS is an appropriate and well-established tool for monitoring of acute recovery-stress states in young athletes (Nässi et al., 2017). Our data showed that ARSS recovery dimensions significantly decreased from morning to evening training sessions while ARSS stress dimensions increased. Our results are in line with previous work from Kölling et al. (2015) and Hitzschke et al. (2016) who showed that ARSS is able to measure recovery and stress imbalances over numerous training days which is why our first research hypothesis can be accepted.

### Resistance Training and Objective Signs

Our results demonstrate highly significant inverse shifts in GRAN_%_ and LYM_%_, due to resistance training in young track and field athletes. These shifts are most likely related to highly significant GRAN (*p* < 0.001) and highly significant WBC (*p* < 0.001) increases from morning to evening training sessions. According to this finding, our second hypothesis can be accepted. Our findings on the acute effects of resistance training are in line with results from Gabriel and Kindermann (1997). These authors observed increases in WBC and GRAN and inverse shifts in GRAN_%_ and LYM_%_ 1–3 h after a single bout of aerobic cycling. In contrast, we were not able to detect decreases in lymphocytes after resistance training. Training type specific lymphocyte kinetics might be related to different metabolic, cardiorespiratory, and hormonal demands in resistance versus endurance training. Even though increases in GRAN are related to improved innate immune function, a reduced bacterial-induced oxidative burst (Robson et al., 1999; Peake and Suzuki, 2004; Nagatomi, 2006) might leave athletes vulnerable to infections (Walsh et al., 2011). We were able to detect changes in capillary blood markers following resistance training which is indicative of exercise-induced immunological responses following this type of training in young track and field athletes.

Previous studies already examined immunological responses following resistance training (Gabriel and Kindermann, 1998; Gleeson et al., 2000; Mayhew et al., 2005; Paulsen et al., 2010; Moreira et al., 2013; Ihalainen et al., 2014; Moraes et al., 2017). However, most studies did not assess immune cell kinetics by means of blood analyses (Paulsen et al., 2010; Moreira et al., 2013; Ihalainen et al., 2014; Moraes et al., 2017). Those few studies that examined immune cell kinetics scrutinized different cohorts and applied different types of resistance training (Mayhew et al., 2005; Ihalainen et al., 2014, 2017). Given that our study sample conducted training in small (relation of supervisor to coach) training groups (see Supplementary Material) with different emphasis, overall comparison of our findings with results from previous studies is hardly feasible.

### Relation of the Acute Recovery and Stress Scale With Capillary Blood Markers

The univariate regression revealed that ARSS stress dimensions are either positively (WBC, MID, GRAN, GRAN_%_) or negatively (LYM_%_ and MID_%_) related to immunological blood markers. The positive association of WBC, GRAN, and GRAN_%_ is in line with the predicted cell kinetics of biphasic leukocytosis (Gabriel and Kindermann, 1997). During the first phase, exercise-induced stress causes migration of WBC (GRAN, LYM, and MID) from the marginal pool into the circulating blood (Gabriel and Kindermann, 1998). This reaction matches specific immune cell kinetics for infections (Northoff et al., 1998). Although our study focused on the investigation of post-training immune cell kinetics, this mechanism may still contribute and explain our findings. The second phase is characterized by further increases of GRAN caused by mobilization of GRAN from the bone marrow and their extended stay within blood circulation. In contrast, LYM is reduced by homing (Gabriel, 2006). Therefore, positive (GRAN, GRAN_%_, and WBC) and negative (LYM_%_ and MID_%_) associations with ARSS stress dimensions may indicate the beginning of the second phase of biphasic leukocytosis. Furthermore, ARSS recovery dimensions showed positive (LYM, LYM_%_, MID_%_, RBC, HGB, HCT) and negative (WBC, GRAN, GRAN_%_ and RDWa) associations with blood measures. Post training and during recovery, a restoring of the homeostasis takes place. Thus, inverse associations of immune parameters could be expected. Positive correlations of the blood markers RBC, HGB, and HCT could also be indicative of an ongoing regeneration process. To elucidate the main determinants responsible for changes during recovery, related blood measures were entered into our multivariate regression analyses. Notably, GRAN_%_ and LYM_%_ were not entered into a joint model due to high multicollinearity. Multivariate models revealed that LYM_%_, GRAN_%_, and WBC accounted for changes in the examined ARSS dimensions. Most importantly, immune measures were identified that are related to changes in the stress-recovery balance. The absolute immune measures were not entered in the joint models though.

As percentage shares of WBC seem to reflect changes in ARSS, *post hoc* analyses were applied to identify the specific directions in relative immune cell shifts in relation to ARSS changes (see **Figure 1**). Our findings emphasized the importance of GRAN_%_ and LYM_%_ for the observed changes in ARSS stress and recovery dimensions. As illustrated in **Figure 1A**, participants with a training-induced decrease in LYM_%_ (41/56 datasets) showed significantly higher reductions in ARSS recovery dimensions and increases in ARSS stress dimensions (**Figure 1B**). In addition, subjects with training-induced increases in GRAN_%_ (41/56 datasets) showed significantly lower ARSS recovery dimensions (**Figure 1C**) and significantly higher ARSS stress dimensions (**Figure 1D**). These findings are in line with the behavior of immune cell kinetics following endurance training. In other words, higher inverse shifts in WBC percentage shares are expressed through changes in the stress-recovery balance using ARSS dimensions. Having this in mind, we can accept our third research hypothesis. Thus, it can be postulated that subjectively measured symptoms (ARSS) of immunological responses following resistance training are associated with objectively tested blood markers (shift of GRAN_%_ and LYM_%_) in young track and field athletes.

### Limitations

Our study was carried out as an observational study that was conducted in the field. Therefore, this study comes with a few limitations that warrant discussion. The most notable limitation of the present study was the narrow post-training window in which capillary blood samples were taken and the subjective measures of acute recovery and stress. However, it has to be noted that the study was designed as an applied study in the field. In contrast to laboratory-based research, our post-training capillary blood samples were not obtained at distinct time points after the respective training sessions. In fact, we had to cope with a delay of 30–45 min post-training. In this regard, it has previously been purported that later measurements are associated with ongoing shifts in GRAN_%_ and LYM_%_ as well as with a decrease in LYM (McCarthy and Dale, 1988; Gabriel and Kindermann, 1997; Gabriel, 2006). However, we would like to point out that the detection of peak immunological responses in WBC, GRAN, and LAM was not the primary goal of this study. Yet, we acknowledge that different post-training time intervals are a limitation of this study. There is evidence of further increases in GRAN_%_ and LYM_%_ shifts during later recovery stages (McCarthy and Dale, 1988; Gabriel and Kindermann, 1997; Gabriel, 2006). In our field setting with young athletes, it was not feasible to take measurements at later time points because this would have affected athletes’ daily training program and schedule (school, social contacts, family). Data from this study do not allow to determine resistance training specific dose-response relationships in the context of training-induced changes in subjective symptoms and objective signs of immunological responses. Well controlled laboratory studies are needed to elucidate this research question.

It has to be acknowledged that ARSS is limited to rating biases. However, self-reported measures using questionnaires represent the most common form of athlete monitoring on an elite level, and are often favored over physiological and performance measures due to cost effectiveness and practical advantages (Taylor et al., 2012). Our study is the first study that investigated associations between changes in subjective symptoms and objectively measured signs of stress and recovery following resistance training. Therefore, when interpreting our findings, it has to be taken into account that research in elite athletes always comes with certain limitations that are specific to the field setting. Finally, we examined young track and field athletes with a mean age of 16.4 years. Caution is needed when translating these findings to other sports, training types, or cohorts.

### Recommendations for the Assessment of Exercise-Induced Immunological Responses

We were able to show for the first time that demands during resistance training are related to immunological responses in young athletes. What practically relevant recommendations can be provided for practitioners working with young athletes? First, as was already shown for endurance training, resistance training is associated with training-induced immunological responses in adolescent young track and field athletes. Thus, daily monitoring (early in the morning and in the evening) of subjective and objective measures of immunological responses for at least 48 h (Spence et al., 2007) and up to 7 days (own data) is recommended during intense training periods. Second, with reference to the identified associations between ARSS and blood markers, ARSS is recommended to be implemented in daily training routines to monitor subjective signs of immunological responses. Third, clinical blood reference values should not be used to determine exercise-induced immunological responses. Even though we were able to show immunological responses following resistance training in young track and field athletes, capillary blood markers did not exceed the reported clinical ranges.

### Future Implications

In order to detect resistance training-induced immunological stress responses in young athletes, the following aspects should be considered for future research:

First, LYM_%_, GRAN_%_ and WBC were related to changes in ARSS stress and recovery dimensions. It has to be noted that even though these blood markers changed significantly due to training, they did not exceed the clinically relevant range. However, to the best of our knowledge, our assessment of changes in subjective symptoms and objective signs during a 7-day resistance training period is a new approach that was not yet tested in a clinical setting. From this it follows that we cannot deduce clinical implications from our findings. Therefore, future research is needed to examine larger cohorts of young athletes and to extend our approach concerning infections (e.g., upper respiratory tract infections). To differ between training-induced and infection-induced immunological responses, cohort specific reference value studies have to be carried out. Clinically relevant immunological changes of subjective symptoms and objective signs concerning resistance training and how they are related to clinically relevant immunological changes (e.g., upper respiratory tract infection) should be addressed in further studies. Data from our study could be used as a first (preliminary) benchmark for the assessment of norm values concerning resistance training-induced immunological responses. Second, associations between ARSS recovery and stress dimensions and objectively measured capillary blood need further validation on larger sample sizes and in adolescent athlete populations. Based on our study findings, future research might concentrate on the detection of immunological responses to resistance training using the ARSS stress and recovery scales. Third, an assessment protocol needs to be established that is easy-to-administer and not time consuming. The practicability should be regarded as a main requirement for future application in young athletes.

## Conclusion

Findings from this study suggest that resistance training is associated with exercise-induced immunological responses in young male and female track and field athletes. More specifically, results from capillary blood samples indicated an increase in WBC, GRAN, GRAN_%_ and a decrease in LYM_%_ from morning to evening training sessions. Daily change-scores of subjective symptoms of recovery and stress dimensions were assessed using ARSS. Accordingly, we observed a concomitant decrease in ARSS recovery dimensions together with an increase in ARSS stress dimensions from morning to evening training sessions over the 7-day study period. Moreover, ARSS scale findings were associated with specific changes in objectively measured immunological markers (GRAN_%_ and LYM_%_) It is noteworthy that participants with an increase in GRAN_%_ also showed a larger decrease in ARSS recovery dimensions and a larger increase in ARSS stress dimensions from morning to evening training sessions. Furthermore, young athletes who showed a decrease in LYM_%_ also demonstrated a larger decrease in ARSS recovery dimensions and a larger increase in ARSS stress dimensions from morning to evening training sessions. Our findings indicate that subjective parameters of stress and recovery using ARSS are suitable to detect resistance-training induced immunological changes over a time period of 7 days. Future well-controlled studies are crucial to determine dose-response relationships following resistance training in young athletes in the context of training-induced immunological responses using subjective (e.g., ARSS) and objective (e.g., blood samples) testing tools.

## Author Contributions

CP, TS, RM, and UG designed the experiments. RM, SW, MH, and CP gathered the data. PB, TS, TW, and CP conducted the data analysis. CP, TS, MK, PB, UG, and HG wrote the manuscript. All authors discussed the results and its implications, commented and edited the manuscript at all stages, and approved the final version.

## Conflict of Interest Statement

The authors declare that the research was conducted in the absence of any commercial or financial relationships that could be construed as a potential conflict of interest.
