# Supplementary material for: Standardized Assessment of Resistance Training-Induced Subjective Symptoms and Objective Signs of Immunological Stress Responses in Young Athletes
Source: Front Physiol. 2018 Jun 5;9:698. doi: 10.3389/fphys.2018.00698 (PMC5996067; doi:10.3389/fphys.2018.00698)
Supplement: Supplementary file 1 [file Table_1.PDF]

Team short sprint:	KS001			
12.CW	21.-27.03.2016	aVP II 4/6	Release 1/1	
**Monday**	**Tuesday**	**Wednesday**	**Thursday**	**Friday**
		**Power 3x10 repetitions**<br>Bench press<br>Bench pull<br>Deadlift<br>Bench press eccentric<br>Squad single leg (per leg)<br>Hip flexors (fast device)		
**AAT**<br>AW Medball 200x<br>Stability 10min<br>6x20m Toe walking barefoot<br><br>**16:15 Uhr**<br>Yoga 30min	**Coordination/Agility**<br>Agility ladder<br><br>**Acceleration**<br>5x 30m ZWL<br>5x 30m sprint submax.<br><br>**Tempo extensive**<br>6x100m<br>P: 30s			

Team short sprint:	KS009 / KS010			
12.CW	21.-27.03.2016	aVP II 4/6	Release 1/1	
**Monday**	**Tuesday**	**Wednesday**	**Thursday**	**Friday**
	**Coordination/Agility**<br>Agility ladder<br><br>**Acceleration**<br>5x 30m ZWL<br>5x 30m sprint submax.<br><br>**Tempo extensive**<br>6x100m<br>P: 30s			
**AAT**<br>AW Medball 200x<br>Stability10min<br>6x20m Toe walking barefoot<br><br>**15:45 Uhr**<br>Yoga 30min		**Power 3x10 repetitions**<br>Bench press<br>Bench pull<br>Dead lift<br>Bench press eccentric<br>Squad single leg (per leg)<br>Hip flexors (fast device)		

Team hurdles:	KS005			
12.CW	21.-27.03.2016	aVP II 4/6	Release 1/1	
**Monday**	**Tuesday**	**Wednesday**	**Thursday**	**Friday**
		**Power 3x10 repetitions**<br>Bench press<br>Bench pull<br>Dead lift<br>Bench press eccentric<br>Squad single leg (per leg)<br>Hip flexors (fast device)		
**AAT**<br>AW Medball 200x<br>Stability 10min<br>6x20m Toe walking barefoot<br><br>**16:15 Uhr**<br>Yoga 30min	**Coordination/Agility**<br>Agility ladder<br><br>**Acceleration**<br>5x 30m ZWL<br>5x 6 hurdles (Distance 24 Feet)<br><br>**Tempo extensive**<br>6x100m<br>P: 30s			

Team hurdles:	KS007 / KS008 / KS012			
12.CW	21.-27.03.2016	aVP II 4/6	Release 1/1	
**Monday**	**Tuesday**	**Wednesday**	**Thursday**	**Friday**
	**Coordination/Agility**<br>Agility ladder<br><br>**Acceleration**<br>5x 30m ZWL<br>5x 6 hurdles (distance 24 Feet)<br><br>**Tempo extensive**<br>6x100m<br>P: 30s			
**AAT**<br>AW Medball 200x<br>Stability 10min<br>6x20m Toe walking barefoot<br><br>**15:45 Uhr**<br>Yoga 30min		**Power 3x10 repetitions**<br>Bench press<br>Bench pull<br>Dead lift<br>Bech press eccentric<br>Squad single leg (per leg)<br>Hip flexors (fast device)		

Team long sprint:	KS003 / KS018			
12.CW	21.-27.03.2016	aVP II 4/6	Release1/1	
**Monday**	**Tuesday**	**Wednesday**	**Thursday**	**Friday**
		**Power 3x10 repetitions**<br>Bench press<br>Bench pull<br>Dead lift<br>Bench press eccentric<br>Squad single leg (per leg)<br>Hip flexors (fast device)		
**AAT**<br>AW Medball 200x<br>Stability 10min<br>6x20m Toe walking barefoot<br><br>**16:15 Uhr**<br>Yoga 30min	**Coordination/Agility**<br>Agility ladder<br><br>**Speed endurance**<br>1-2-3-4-1-2-300m 85%<br>P: 4/6/8/12/4/6min<br><br>*JG: 12,4/25,8/43,2/60,3*<br>*MO: 14,0/29,6/50,0/68,5*			

Team long sprint:	KS002 / KS004 / KS020			
12.CW	21.-27.03.2016	aVP II 4/6	Release 1/1	
**Monday**	**Tuesday**	**Wednesday**	**Thursday**	**Friday**
	**Coordination/Agility**<br>Agility ladder<br><br>**Speed endurance**<br>1-2-3-4-1-2-300m 85%<br>P: 4/6/8/12/4/6min<br><br>*LP: 12,9/26,6/43,2/60,1*<br>*JU: 12,2/25,6/42,7/59,5*<br>*AR: 14,7/30,3/50,3/68,9*			
**AAT**<br>AW Medball 200x<br>Stability 10min<br>6x20m Toe walking barefoot<br><br>**15:45 Uhr**<br>Yoga 30min		**Power 3x10 repetitions**<br>Bench press<br>Bench pull<br>Dead lift<br>Bench press eccentric<br>Squad single leg (per leg)<br>Hip flexors (fast device)		

Team long jump:	KS016			
**12.CW**	**21.-27.03.2016**	**aVP II 4/6**	**Release 1/1**	
**Monday**	**Tuesday**	**Wednesday**	**Thursday**	**Friday**
		**Power 3x10 repetitions**<br>Bench press<br>Bench pull<br>Dead lift<br>Bench press eccentric<br>Squad single leg (per leg)<br>Hip flexors (fast device)		
**AAT**<br>AW Medball 200x<br>Stability 10min<br>6x20m Toe walking barefoot<br><br>**16:15 Uhr**<br>Yoga 30min	**Coordination/Agility**<br>Agility ladder<br><br>**Acceleration**<br>5x 30m ZWL<br>5x 6 hurdles (distance 24 Feet)<br><br>**Jump power vertical**<br>10x 10 ankle jumps intensiv<br>P: 1:30min			

Team long jump:	KS014			
**12.CW**	**21.-27.03.2016**	**aVP II 4/6**	**Release 1/1**	
**Monday**	**Tuesday**	**Wednesday**	**Thursday**	**Friday**
	**Coordination/Agility**<br>Agility ladder<br><br>**Acceleration**<br>5x 30m ZWL<br>5x 6 hurdles (Distance 24 Feet)<br><br>**Jump power vertical**<br>10x 10 ankle jumps intensiv<br>P: 1:30min			
**AAT**<br>AW Medball 200x<br>Stability 10min<br>6x20m Toe walking barefoot<br><br>**15:45 Uhr**<br>Yoga 30min		**Power 3x10 repetitions**<br>Bench press<br>Bench pull<br>Dead lift<br>Bench press eccentric<br>Squad single leg (per leg)<br>Hip flexors (fast device)		

9th/10th grade
Saturday
Sunday

11th/12th grade
Saturday
Sunday

9th/10th grade
Saturday
Sunday

11th/12th grade
Saturday
Sunday

9th/10th grade
Saturday
Sunday

11th/12th grade
Saturday
Sunday

10th grade
Saturday
Sunday

11th/12th grade
Saturday
Sunday
