# Supplementary material for: Standardized Assessment of Resistance Training-Induced Subjective Symptoms and Objective Signs of Immunological Stress Responses in Young Athletes
Source: Front Physiol. 2018 Jun 5;9:698. doi: 10.3389/fphys.2018.00698 (PMC5996067; doi:10.3389/fphys.2018.00698)
Supplement: Supplementary file 2 [file Table_2.PDF]

Team short sprint:	KS001				9th/10th grade
13.CW	28.03.-03.04.2016	aVP II 5/6	Load 1/3		
**Monday**	**Tuesday**	**Wednesday**	**Thursday**	**Friday**	**Saturday**
	**Coordination/Agility**<br>Sprint-ABC<br><br>**Sprint power/Acceleration**<br>5x<br>30m ZWL/30m Sprint<br>in alternation	**AAT**<br>jump power horizontal 5x20m:<br>Alternating lunge jumps<br>High knee skips<br>Jump run<br>Snatch (5x10m)<br><br>**Tempo extensive**<br>10x100m<br>P: 30s	**Coordination**<br><br>**Speed**<br>4x 60m increments ZWL; P: 5min<br>4x 30m flying submax.; P: 5-6min<br><br>**AW Bowl**<br>Shock fw/bw je 10x	**3x increments 50m**<br><br>**Speed endurance (curve)**<br>2x 80/100/120 90%<br>P: 6/8/10min<br><br>**AAT**<br>Stability 20min	**TEST**<br>Shock forwards<br>Shock backwards<br>standing long jump<br>5er SPL with run-up
Warm up run 10min<br>Gymnastic 10min<br>Cool down run 10min	**Power**	**FREE**	**Power**	**FREE**	**AAT**<br>Minibands<br>Jump power vertical 5x20 rep<br>Ankle jumps<br>Squad jumps<br>Scissor jumps<br>Metcalf<br><br>**Tempo extensive**<br>10x100m ; P:30s<br><br>**Sonday**<br><br>**FREE**

Team hurdles:	KS005 / KS016				9th/10th grade
13.CW	28.03.-03.04.2016	aVP II 5/6	Load 1/3		
**Monday**	**Tuesday**	**Wednesday**	**Thursday**	**Friday**	**Saturday**
	**Coordination**<br>Hurdles-ABC<br><br>**Sprint power/Acceleration**<br>4x<br>30m ZWL/ 2. Hurdle<br>in alteration	**AAT**<br>jump power horizontal 5x20m:<br>Alternating lunge jumps<br>High knee skips<br>Jump run<br>Snatch (5x10m)<br><br>**Tempo extensive**<br>10x100m<br>P: 30s	**Coordination**<br>short 3er, 5 hurdles<br>short 1er, 5 hurdles<br><br>**Hurdles**<br>6x 10 hurdles 3er R.<br>Distance 7,50m<br><br>**AW Bowl**<br>Shock fw/bw 10x each	**3x increments 50m**<br><br>**Speed endurance (curve)**<br>2x 80/100/120 90%<br>P: 6/8/10min<br><br>**AAT**<br>Stability 20min	**TEST**<br>Shock forwards<br>Shock backwards<br>Standing long jump<br>5er SPL with run-up
Warm up run 10min<br>Gymnastic 10min<br>Cool down run 10min	**Power**	**Free**	**Power**	**FREE**	**AAT**<br>Minibands<br>Jump power vertical 5x20 rep<br>Ankle jumps<br>Squad jumps<br>Scissor jumps<br>Metcalf<br><br>**Tempo extensive**<br>10x100m ; P:30s
					**Sunday**
					**FREE**

Team hurdles:	KS007 / KS008 / KS012 / KS014				11th/12th grade
13.CW	28.03.-03.04.2016	aVP II 5/6	Load 1/3		
**Monday**	**Tuesday**	**Wednesday**	**Thursday**	**Friday**	**Saturday**
	**Coordination**<br>Hurdles-ABC<br><br>**Sprint power/Acceleration**<br>5x<br>30m ZWL/ 2. Hurdle<br>in alteration	**AAT**<br>Jump power horizontal 5x20m:<br>Alternating lunge jumps<br>High knee skips<br>Jump run<br>Snatch (5x10m)<br><br>**Tempo extensive**<br>10x100m<br>P: 30s	**Coordination**<br>short 3er, 5 hurdles<br>short 1er, 5 hurdles<br><br>**Hurdles**<br>8x 10 hurdles 3er R.<br>Distance 7,50m<br><br>**AW Bowl**<br>Shock fw/bw je 10x	**3x increments 50m**<br><br>**Speed endurance (curve)**<br>2x 80/100/120 90%<br>P: 6/8/10min<br><br>**AAT**<br>Stability 20min	**TEST**<br>Shock forwards<br>Shock backwards<br>Standing long jump<br>5er SPL with run-up
Warm up run 10min<br>Gymnastic 10min<br>Cool down run 10min	**Power**	**FREE**	**Power**	**FREE**	**AAT**<br>Minibands<br>Jump power vertical 5x20 rep<br>Ankle jumps<br>Squad jumps<br>Scissor jumps<br>Metcalf<br><br>**Tempo extensive**<br>10x100m ; P:30s
					**Sunday**
					**FREE**

Team long sprint:	KS003 / KS018				9th/10th grade
13.CW	28.03.-03.04.2016	aVP II 5/6	Load 1/3		
**Monday**	**Tuesday**	**Wednesday**	**Thursday**	**Friday**	**Saturday**
	**Coordination**<br>Sprint-ABC<br><br>**Jump power/Acceleration**<br>4x<br>30m ZWL/30m Sprint<br>in alternation	**AAT**<br>Jump power horizontal 5x20m:<br>Alternating lunge jumps<br>High knee skips<br>Jump run<br>Snatch (5x10m)<br><br>**Tempo extensive**<br>10x200m<br>P: 1min	**Coordination**<br><br><br>**Speed**<br>4x 60m increments ZWL; P: 5min<br>4x 30m flying submax.; P: 5-6min<br><br><br>**AW Bowl**<br>Shock fw/bw 10x each	**Hurdles**<br>5x 5 hurdles 5er R. curve<br><br><br><br><br>**Speed endurance**<br>2x 100/200/300m 85%<br>P: 4/6/8min<br><br>**AAT**<br>Stability 20min	**TEST**<br>Shock forwards<br>Shock backwards<br>Standing long jump<br>5er SPL with run-up
Warm up run 10min<br>Gymnastic 10min<br>Cool down run 10min	**Power**		**Power**		**AAT**<br>Minibands<br>Jump power vertical 5x20 rep<br>Ankle jumps<br>Squad jumps<br>Scissor jumps<br>Metcalf<br><br>**Tempo extensiv**<br>10x100m ; P:30s<br>**Sunday**<br><br>**FREE**

Team long sprint:	KS002 / KS004 / KS020				11th/12th grade
13.CW	28.03.-03.04.2016	aVP II 5/6	Load 1/3		
**Monday**	**Tuesday**	**Wednesday**	**Thursday**	**Friday**	**Saturday**
	**Coordination**<br>Sprint-ABC<br><br>**jump power/Acceleration**<br>5x<br>30m ZWL/30m Sprint<br>in alternation	**AAT**<br>Jump power horizontal 5x20m:<br>Alternating lunge jumps<br>High knee skips<br>Jump run<br>Snatch (5x10m)<br><br>**Tempo extensive**<br>10x200m<br>P: 1min	**Coordination**<br><br><br>**Speed**<br>4x 60m increments ZWL; P: 5min<br>4x 30m flying submax.; P: 5-6min<br><br><br>**AW Bowl**<br>Shock fw/bw 10x each	**Hurdles**<br>5x 5 hurdles 5er R. curve<br><br><br><br><br>**Speed endurance**<br>2x 100/200/300m 85%<br>P: 4/6/8min<br><br>**AAT**<br>Stability 20min	**TEST**<br>Shock forwards<br>Shock backwards<br>Standing long jump<br>5er SPL with run-up
Warm up run 10min<br>Gymnastic 10min<br>Cool down run 10min	**Power**		**Power**		**AAT**<br>Minibands<br>Jump power vertical 5x20 rep<br>Ankle jumps<br>Squad jumps<br>Scissor jumps<br>Metcalf<br><br>**Tempo extensive**<br>10x100m ; P:30s<br>**Sunday**<br><br>**FREE**

Team long jump:	KS016				10th grade
13.CW	28.03.-03.04.2016	aVP II 5/6	Load 1/3		
**Monday**	**Tuesday**	**Wednesday**	**Thursday**	**Friday**	**Saturday**
					**Sunday**
Warm up run 10min<br>Gymnastic 10min<br>Cool down run 10min					

Team long jump:	KS014				11th/12th grade
13.CW	28.03.-03.04.2016	aVP II 5/6	Load 1/3		
**Monday**	**Tuesday**	**Wednesday**	**Thursday**	**Friday**	**Saturday**
					**Sunday**
Warm up run 10min<br>Gymnastic 10min<br>Cool down run 10min					

s.:

s.:

s.:

s.:

s.:
