# Supplementary material for: Standardized Assessment of Resistance Training-Induced Subjective Symptoms and Objective Signs of Immunological Stress Responses in Young Athletes
Source: Front Physiol. 2018 Jun 5;9:698. doi: 10.3389/fphys.2018.00698 (PMC5996067; doi:10.3389/fphys.2018.00698)
Supplement: Supplementary file 3 [file Table_3.PDF]

4x 10m block start submax.

**Team short sprint:**	**KS001**				**9th/10th grade**
**14.CW**	**04.-10.04.2016**	**aVP II 6/6**	**Load 2/3**		
**Monday**	**Tuesday**	**Wednesday**	**Thursday**	**Friday**	**Saturday**
**Coordination**<br><br>**Sprint power/Acceleration**<br>4x 30m ZWL	**Coordination**<br><br>**Speed endurance**<br>2x120/150/200m 90%<br>P: 12/15/20min	**Departure**			
**Power**	**AAT**<br>Minibands<br>Jump power vertical 5x20 reps.:<br>Ankle jumps<br>Squad jumps<br>Scissor jumps<br>Metcalf<br><br>**Tempo extensive**<br>10x100m ; P:30s				**Sunday**

Team hurdles:	KS005 / KS016				9th/10th grade
14.CW	04.-10.04.2016	aVP II 6/6	Load 2/3		
**Monday**	**Tuesday**	**Wednesday**	**Thursday**	**Friday**	**Saturday**
**Coordination**<br>Wall Drill<br>Lead leg/trail leg<br>je 3x 10 per side<br><br>**Hurdles**<br>6x 5 hurdles	**Coordination**<br><br>**Speed endurance**<br>2x120/150/200m 90%<br>P: 12/15/20min	**Departure**			
**Power**	**AAT**<br>Minibands<br>Jump power vertical 5x20 reps.:<br>Ankle jumps<br>Squad jumps<br>Scissor jumps<br>Metcalf<br><br>**Tempo extensive**<br>10x100m ; P:30s				**Sonntag**

Team hurdles:	KS007 / KS008 / KS012 / KS014				11th/12th grade
14.CW	04.-10.04.2016	aVP II 6/6	Load 2/3		
**Monday**	**Tuesday**	**Wednesday**	**Thursday**	**Friday**	**Saturday**
**Coordination**<br>Wall Drill<br>Lead leg/trail leg<br>je 3x 10 per side<br><br>**Hurdles**<br>6x 5 hurdles	**Coordination**<br><br>**Speed endurance**<br>2x120/150/200m 90%<br>P: 12/15/20min	**Departure**			
**Power**	**AAT**<br>Minibands<br>Jump power vertical 5x20 reps.:<br>Ankle jumps<br>Squad jumps<br>Scissor jumps<br>Metcalf<br><br>**Tempo extensive**<br>10x100m ; P:30s				**Sunday**

Team long sprint:	KS003 / KS018				9th/10th grade
**14.CW**	**04.-10.04.2016**	**aVP II 6/6**	**Load 2/3**		
**Monday**	**Tuesday**	**Wednesday**	**Thursday**	**Friday**	**Saturday**
**Coordination**<br><br>**Sprint power/ Acceleration**<br>4x 30m ZWL<br>4x 10m block start submax.	**Coordination**<br><br>**Speed endurance**<br>2x120/150/200m 90%<br>P: 12/15/20min	**Departure**			
**Power**	**AAT**<br>Minibands<br>Jump power vertical 5x20 reps.:<br>Ankle jumps<br>Squad jumps<br>Scissor jumps<br>Metcalf<br><br>**Tempo extensive**<br>10x100m ; P:30s				**Sunday**

Team long sprint:	KS002 / KS004 / KS020				11th/12th grade
**14.CW**	**04.-10.04.2016**	**aVP II 6/6**	**Load 2/3**		
**Monday**	**Tuesday**	**Wednesday**	**Thursday**	**Friday**	**Saturday**
**Coordination**<br><br>**Sprint power/Acceleration**<br>4x 30m ZWL<br>4x 10m block start submax.	**Coordination**<br><br>**Speed endurance**<br>2x120/150/200m 90%<br>P: 12/15/20min	**Departure**			
**Power**	**AAT**<br>Minibands<br>Jump power vertical 5x20 reps.:<br>Ankle jumps<br>Squad jumps<br>Scissor jumps<br>Metcalf<br><br>**Tempo extensive**<br>10x100m ; P:30s				**Sunday**

Team long jump:	KS016				10th grade
14.CW	04.-10.04.2016	aVP II 6/6	Load 2/3		
Monday	Tuesday	Wednesday	Thursday	Friday	Saturday
Warm up run 10min<br>Gymnastic 10min<br>Cool down run 10min					Sunday

Team long jump:	KS014				11th/12th grade
14.CW	04.-10.04.2016	aVP II 6/6	Load 2/3		
Monday	Tuesday	Wednesday	Thursday	Friday	Saturday
Warm up run 10min<br>Gymnastic 10min<br>Cool down run 10min					Sunday
