# Supplementary material for: Standardized Assessment of Resistance Training-Induced Subjective Symptoms and Objective Signs of Immunological Stress Responses in Young Athletes
Source: Front Physiol. 2018 Jun 5;9:698. doi: 10.3389/fphys.2018.00698 (PMC5996067; doi:10.3389/fphys.2018.00698)
Supplement: Supplementary file 4 [file Table_4.PDF]

Team short sprint:	KS001				9th/10th grade
15.CW	11.-17.04.2016	sVP II 1/7	Load 3/3		
**Monday**	**Tuesday**	**Wednesday**	**Thursday**	**Friday**	**Saturday**
		**Power 3x8 reps.**<br>Bench press<br>Bench pull<br>Dead lift<br>Bench press eccentric<br>Squad single leg (per leg)<br>Hip flexors (fast device)		**Power 3x8 reps.**<br>Lat pull-down<br>Calf raises<br>Transpose<br>Squads (deep)<br>Step-up (high)<br>Leg flexors skateboard	
					**Sunday**
**AAT**<br>Stabibiliy 20min<br><br>**Tempo extensive**<br>10x 120m (lawn diagonal)<br>*walking pause*	**Coordination**<br><br>**Sprint power/Acceleration**<br>3x 30m ZWL 5%, P: 3min<br>5x 20m 3-P-Start, P: 2min<br><br>**Speed endurance**<br>3x 90m TW (30/30/30), P: 4min<br>3x 120m TW (40/40/40) P: 6min		**Coordination**<br>Single leg run<br><br>**Speed**<br>change in frequency<br>3x 10/10/10/10/10/10m<br>3x 20/10/20/10<br>P: je 5-6min<br><br>**Specific endurance**<br>4x 150m Build-up (50/50/50)<br>P: 10min	*15:00 Uhr*<br>*TLV-Workshop*	

Team short sprint:	KS009 / KS010				11th/12th grade
15.CW	11.-17.04.2016	sVP II 1/7	Load 3/3		
**Monday**	**Tuesday**	**Wednesday**	**Thursday**	**Friday**	**Saturday**
	**Coordination**<br><br>**Sprint power/Acceleration**<br>3x 30m ZWL 5%, P: 3min<br>5x 20m 3-P-Start, P: 2min<br><br>**Speed endurance**<br>3x 90m TW (30/30/30), P: 4min<br>3x 120m TW (40/40/40) P: 6min	**Power 3x8 reps.**<br>Bench press<br>Bench pull<br>Dead lift<br>Bench press eccentric<br>Squad single leg (per leg)<br>Hip flexors (fast device)			
					**Sunday**
**AAT**<br>Stability 20min<br><br>**Tempo extensive**<br>10x 120m (lawn diagonal)<br>*walking pause*			**Coordination**<br>Single leg run<br><br>**Speed**<br>change in frequency<br>3x 10/10/10/10/10/10m<br>3x 20/10/20/10<br>P: je 5-6min	**Eleni:**<br><br>**Specific endurance**<br>2x 250m 85% , P: 10min<br>2x 200m 85%, P: 10min<br><br>**Lisa:**<br><br>**Speed endurance**<br>4x 120m 90%, P: 6min	

Team hurdles:	KS005				9th/10th grade
15.CW	11.-17.04.2016	sVP II 1/7	Load 3/3		
**Monday**	**Tuesday**	**Wednesday**	**Thursday**	**Friday**	**Saturday**
		**Power 3x8 reps.**<br>Bench press<br>Bench pull<br>Dead lift<br>Bench press eccentric<br>Squad single leg (per leg)<br>Hip flexors (fast device)		**Power 3x8 reps.**<br>Lat pull-down<br>Calf raises<br>Transpose<br>Squads (deep)<br>Step-up (high)<br>Leg flexors Skateboard	
					**Sunday**
**AAT**<br>Stabiility 20min<br><br>**Tempo extensive**<br>10x 120m (lawn diagonal)<br>*walking pause*	**Hurldes Coordination**<br>Hurdle-ABC<br><br>**Sprint power/Hurdles acceleration**<br>3x 30m ZWL 5%, P: 3min<br>5x 2. hurdle 3-P-Start P: 3min<br><br>**Speed endurance**<br>3x 90m TW (30/30/30), P: 4min<br>3x 120m TW (40/40/40) P: 6min		**Hurldes Coordination**<br>Enter 5x 5 hurdles<br><br>**Hurdles speed**<br>4x 10. hurdles (Distance 7,50m)<br>P: 6-8min<br><br>**Specific endurance**<br>2-3x 150m Build-up (50/50/50)<br>P: 10min	*15:00 Uhr*<br>*TLV-Workshop*	

Team hurdles:	KS007 / KS008 / KS012				11th/12th grade
15.CW	11.-17.04.2016	sVP II 1/7	Load 3/3		
**Monday**	**Tuesday**	**Wednesday**	**Thursday**	**Friday**	**Saturday**
	**Hurldes Coordination**<br>Hurdle-ABC<br><br>**Sprint power/Hurdles acceleration**<br>3x 30m ZWL 5%, P: 3min<br>5x 2. hurdle 3-P-Start P: 3min<br><br>**Speed endurance**<br>3x 90m TW (30/30/30), P: 4min<br>3x 120m TW (40/40/40) P: 6min		**Hurldes Coordination**<br>Enter 5x 5 hurdles<br><br>**Hurdles speed**<br>4x 10. hurdles (Distance 8m/7,50m)<br>P: 6-8min<br><br>**Specific endurance**<br>3x 150m Build-up (50/50/50)<br>P: 10min		
					**Sunday**
**AAT**<br>Stabiliity 20min<br><br>**Tempo extensive**<br>10x 120m (lawn diagonal)<br>*walking pause*	**Power 3x8 reps.**<br>Bench press<br>Bench pull<br>Dead lift<br>Bench press eccentric<br>Squad single leg (per leg)<br>Hip flexors (fast device)		**Power 3x8 reps.**<br>Lat pull-down<br>Calf raises<br>Transpose<br>Squads (deep)<br>Step-up (high)<br>Leg flexors Skateboard	**AAT**<br>Stabiliity 20min<br>Jump power vertikal 4x20 reps.:<br>Ankle jumps<br>Squad jumps<br>Metcalf<br><br>*15:00 Uhr*<br>*TLV-Workshop*	

**Team long sprint:**	**KS003 / KS018**				**9th/10th grade**
**15.CW**	**11.-17.04.2016**	**sVP II 1/7**	**Load 3/3**		
**Monday**	**Tuesday**	**Wednesday**	**Thursday**	**Friday**	**Saturday**
		**Power 3x8 reps.**<br>Bench press<br>Bench pull<br>Dead lift<br>Bench press eccentric<br>Squad single leg (per leg)<br>Hip flexors (fast device)		**Power 3x8 reps.**<br>Lat pull-down<br>Calf raises<br>Transpose<br>Squads (deep)<br>Step-up (high)<br>Leg flexors skateboard	
**AAT**<br>Stability 20min<br><br><br>**Tempo extensive**<br>8x 250m (Lawn-U)<br>*walking pause*	**Coordination**<br><br><br>**Sprint power/Acceleration**<br>3x 30m ZWL 5%, P: 3min<br>5x 20m 3-P-Start, P: 2min<br><br><br>**Speed endurance**<br>5x 120m 90%<br>P: 6min		**Hurldes Coordination**<br>5x 5 hurdles 4er Rhythm<br><br>**Hurdles**<br>2x 1. hurdles<br>2x 2. hurdles<br>2x 3. hurdles<br><br>**Specific endurance**<br>2x 300m 85% (46,9s)<br>P: 10min	 <br>*15:00 Uhr*<br>*TLV-Workshop*	**Sunday**

**Team long sprint:**	**KS002 / KS004 / KS020**				**11th/12th grade**
**15.CW**	**11.-17.04.2016**	**sVP II 1/7**	**Load 3/3**		
**Monday**	**Tuesday**	**Wednesday**	**Thursday**	**Friday**	**Saturday**
	**Coordination**<br><br><br>**Sprint power/Acceleration**<br>3x 30m ZWL 5%, P: 3min<br>5x 20m 3-P-Start, P: 2min<br><br><br>**Speed endurance**<br>5x 120m 90%<br>P: 6min		**Coordination**<br>single leg run<br><br><br>**Speed**<br>change of frequency<br>3x 10/10/10/10/10/10m<br>3x 20/10/20/10<br>P: je 5-6min		
 AAT<br>Stability 20min<br><br><br>**Tempo extensive**<br>8x 250m (Lawn-U)<br>*walking pause*	 **Power 3x8 reps.**<br>Bench press<br>Bench pull<br>Dead lift<br>Bench press eccentric<br>Squad single leg (per leg)<br>Hip flexors (fast device)		**Hurldes Coordination**<br>5x 5 hurdles 4er Rhythm<br><br>**Hurdles**<br>2x 1. hurdles<br>2x 2. hurdles<br>2x 3. hurdles<br><br>**Specific endurance**<br>2x 300m 85% (46,9s)<br>P: 10min	**Power 3x8 reps.**<br>Lat pull-down<br>Calf raises<br>Transpose<br>Squads (deep)<br>Step-up (high)<br>Leg flexors skateboard<br><br><br>*15:00 Uhr*<br>*TLV-Workshop*	**Sunday**

Team long jump:	KS016				10th grade
15.CW	11.-17.04.2016	sVP II 1/7	Load 3/3		
**Monday**	**Tuesday**	**Wednesday**	**Thursday**	**Friday**	**Saturday**
		**Power 3x8 reps.**<br>Bench press<br>Dead lift<br>Squad single leg<br>Hip flexors (fast device)<br><br>**jump power horizontal**<br>5x 30m jump run intensiv<br>3x 30m single leg jump rechts<br>3x 30m single leg jump		**Jump-ABC**<br>8 Exercises<br><br>**Long**<br>8x Take off<br>8x Take off with landing	
					**Sunday**
**AAT**<br>Stability 20min<br><br>**Tempo extensive**<br>10x 120m (lawn diagonal)<br>*walking pause*	**Hurldes Coordination**<br>Hurdles-ABC<br><br>**Sprint power/Hurdle acceleration**<br>3x 30m ZWL 5%, P: 3min<br>5x 2. hurdle 3-P-Start P: 3min<br><br>**Speed endurance**<br>3x 90m TW (30/30/30), P: 4min<br>3x 120m TW (40/40/40) P: 6min		**Hurldes Coordination**<br>Enter 5x 5 hurdles<br><br>**Hurldes speed endurance**<br>4x 10. hurdles (Distance 7,50<br>P: 8min<br><br>**Specific endurance**<br>2-3x 150m Build-up (50/50/50)<br>P: 10min	*15:00 Uhr*<br>*TLV-Workshop*	

Team long jump:	KS014				11th/12th Klasse
15.CW	11.-17.04.2016	sVP II 1/7	Load 3/3		
**Monday**	**Tuesday**	**Wednesday**	**Thursday**	**Friday**	**Saturday**
	**Hurldes Coordination**<br>Hurdles-ABC<br><br>**Sprint power/Hurdle acceleration**<br>3x 30m ZWL 5%, P: 3min<br>5x 2. hurdle 3-P-Start P: 3min<br><br>**Speed endurance**<br>3x 90m TW (30/30/30), P: 4min<br>3x 120m TW (40/40/40) P: 6min		**Hurldes Coordination**<br>Enter 5x 5 hurdles<br><br>**Hurldes speed endurance**<br>4x 10 hurdles (Distance 7,50m)<br>P: 8min<br><br>**Specific endurance**<br>3x 150m Build-up (50/50/50)<br>P: 10min		
					**Sunday**
**AAT**<br>Stability 20min<br><br>**Tempo extensive**<br>10x 120m (lawn diagonal)<br>*walking pause*	**Power 3x8 reps.**<br>Bench press<br>Dead lift<br>Squad single leg<br>Hip flexors (fast device)<br><br>**Jump power horizontal**<br>5x 30m jump run intensiv<br>3x 30m single leg jump rechts<br>3x 30m single leg jump		**Power 3x8 reps.**<br>Lat pull-down<br>Calf raises<br>Transpose<br>Squads (deep)<br>Step-up (high)<br>Leg flexors Skateboard	**Jump-ABC**<br>8 Exercise<br><br>**Long**<br>8x Take off<br>8x Take off with landing<br><br>*15:00 Uhr*<br>*TLV-Workshop*	
